# *Pseudomonas otitidis* bacteremia following endoscopic biliary intervention: A rare case report and review of the literature

**DOI:** 10.1016/j.idcr.2026.e02720

**Published:** 2026-08-07

**Authors:** Takehiro Hashimoto, Ryota Sagami, Yasuhisa Hiroshima, Hiroki Yoshikawa, Kosaku Komiya, Kazufumi Hiramatsu

**Affiliations:** a Hospital Infection Control Center, Oita University Hospital, Oita, Japan; b Department of Gastroenterology, Faculty of Medicine, Oita University, Oita, Japan; c Department of Advanced Gastrointestinal Cancer Medicine, Faculty of Medicine, Oita University, Oita, Japan; d Department of Respiratory Medicine and Infectious Diseases, Oita University Faculty of Medicine, Oita, Japan

**Keywords:** Bacteremia, *Pseudomonas otitidis*, Endoscopic biliary procedures

## Abstract

**Background:**

*Pseudomonas otitidis* is primarily associated with ear infections; invasive disease is exceedingly rare, with only three reported cases of bacteremia in immunocompromised patients. We report the first known case of *P. otitidis* bacteremia following endoscopic biliary procedures.

**Case presentation:**

An 86-year-old woman was admitted for evaluation of elevated levels of hepatobiliary enzymes and intrahepatic bile duct dilation. On the day of admission, endoscopic retrograde cholangiopancreatography complicated by periampullary perforation required conservative management. She developed high-grade fever on day 16. Blood cultures showed growth of gram-negative rods, which were later identified as *P. otitidis* using matrix-assisted laser desorption ionization time-of-flight mass spectrometry and 16S rRNA sequencing. The presumed source of infection was biliary tract infection. Meropenem treatment was initiated because the isolate was phenotypically susceptible to meropenem, and the patient’s condition improved rapidly. Therapy was subsequently switched to ceftazidime, and the patient was discharged without recurrence.

**Conclusion:**

We report a novel case of *P. otitidis* bacteremia following endoscopic biliary procedures. Although *P. otitidis* has been detected in clinical specimens, bacteremia is extremely rare. Clinicians should recognize that *P. otitidis* can cause bacteremia and biliary tract infection.

## Introduction

*Pseudomonas otitidis* was first described in 2006 as a novel species isolated from patients with otitis externa [1]. The organism is considered an uncommon human pathogen with a well-established association with ear infections, including acute otitis externa and chronic otitis media [1], [2]. Infections other than ear infections are rare; however, cases of necrotizing fasciitis, peritonitis, and epididymo-orchitis have been reported [3], [4]. A unique feature of *P. otitidis* is its intrinsic ability to produce the chromosomally encoded metallo-β-lactamase POM−1 (also known as *P. otitidis* MBL−1) [5]. POM−1 hydrolyzes penicillins and carbapenems efficiently but demonstrates low activity against cephalosporins [5].

To date, only three cases of bacteremia caused by *P. otitidis* have been reported in immunocompromised patients [6], [7], [8]. Herein, we report a case of bacteremia following endoscopic biliary procedures.

## Case

An 86-year-old woman was referred to our hospital after routine testing at a previous hospital revealed elevated levels of hepatobiliary enzymes. Abdominal ultrasonography and contrast-enhanced computed tomography revealed intrahepatic bile duct dilation in segment 6 and a 16-mm low-attenuation area adjacent to the portal vein in segment 8, and was admitted for further evaluation. She had a medical history of chronic atrial fibrillation and dyslipidemia and was taking edoxaban and atorvastatin. She had no history of immunosuppressive therapy, alcohol consumption, or smoking. Laboratory tests revealed elevated levels of hepatobiliary enzymes: total bilirubin, 2.0 mg/dL; aspartate aminotransferase, 24 U/L; alanine aminotransferase (ALT), 15 U/L; gamma-glutamyl transpeptidase (γ-GTP), 160 U/L; and alkaline phosphatase, 136 U/L. The level of cancer antigen 19–9 was 1084 U/mL.

**Fig. 1:**
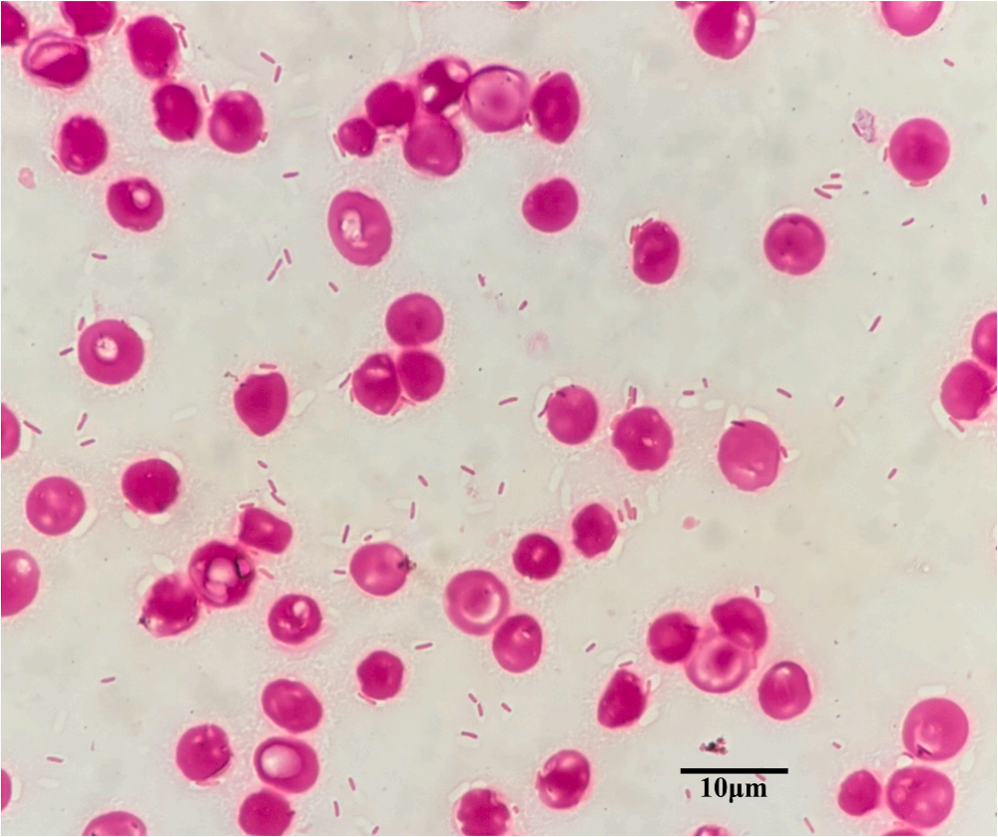
Gram staining performed on the blood cultures reveals gram-negative rods, which appear as a thin, slender rod (×1000 magnification).

On the day of admission, endoscopic retrograde cholangiopancreatography was performed for diagnosis. However, cannulation of the bile duct proved difficult, necessitating endoscopic sphincterotomy and endoscopic papillary balloon dilation. During the procedure, free air was detected around the liver, raising concerns about intraoperative perforation. Endoscopic retrograde cholangiopancreatography for diagnostic purposes was discontinued, and endoscopic nasobiliary drainage (ENBD) was performed. The patient was managed conservatively with bowel rest and initiation of meropenem (MEPM) without cultures. On day 2, laboratory tests revealed an increased C-reactive protein (CRP) level (4.06 mg/dL). On day 3, considering the risk of self-removal associated with ENBD, a biliary stent was placed. On day 7, the CRP level improved (0.69 mg/dL), and MEPM was discontinued. Oral intake was resumed with a liquid diet on day 8, and her clinical course remained stable until she developed fever on day 16. Her body temperature was 40.0 °C, blood pressure was 110/56 mmHg, pulse rate was 120 beats/min, respiratory rate was 22 breaths/min, and oxygen saturation was 98% on room air. She had no cardiac murmurs, her lungs were clear on auscultation, and neither Charcot’s triad nor Murphy’s sign was observed. Other physical examinations revealed no remarkable findings. Laboratory tests revealed a normal white blood cell count (8030/μL) but markedly elevated CRP (13.8 mg/dL), ALT (152 U/L), and γ-GTP (152 U/L) levels. No peripheral or central venous catheter was present. Abdominal computed tomography revealed no abscess, and blood and urine cultures were obtained, with MEPM started empirically. On day 18, her body temperature decreased to 36.8 °C, and the CRP level decreased to 3.61 mg/dL. Urine cultures yielded negative results, whereas two sets of aerobic blood culture bottles showed growth of gram-negative rods (Fig. 1) that were identified as *P. otitidis* using matrix-assisted laser desorption ionization time-of-flight mass spectrometry (Bruker Daltonics, GmbH, Bremen, Germany; score value, 2.16) and 16S rRNA gene sequencing. Total DNA was extracted using Cica Geneus DNA Extraction Kit (Kanto Chemical Co., Inc., Tokyo, Japan) according to the manufacturer’s instructions. The 16S rRNA gene was amplified using the primers 518 F (5′-CCAGCAGCCGCGGTAATAC−3′) and 800 R (5′-TACCAGGGTATCTAATCC−3′). Nucleotide sequencing was conducted using BigDye Terminator v3.1 Cycle Sequencing Kit (Thermo Fisher Scientific, Waltham, MA, USA), and the sequence data were analyzed with SeqStudio Genetic Analyzer (Thermo Fisher Scientific). A Basic Local Alignment Search Tool search (https://blast.ncbi.nlm.nih.gov/) of the obtained sequence data identified the bacterium as *P. otitidis*, showing 99.86% sequence identity (1463/1465 nucleotides) with *P. otitidis* strain NR_043289.1. The patient was diagnosed with *P. otitidis* bacteremia presumably secondary to biliary tract infection.

Antimicrobial susceptibility was assessed according to the Clinical and Laboratory Standards Institute guidelines [9]. The minimum inhibitory concentrations for this isolate, determined using a dry plate (Eiken, Tokyo, Japan) for the broth microdilution method and analyses via an image analyzer (Koden IA60F; Koden, Tokyo, Japan), were as follows: piperacillin, 4 µg/mL; piperacillin/tazobactam, 4/4 µg/mL; ceftolozane/tazobactam, 2/4 µg/mL; ceftazidime, 2 µg/mL; cefepime, 1 µg/mL; aztreonam, 8 µg/mL; meropenem, 1 µg/mL; amikacin, ≤ 0.5 µg/mL; ciprofloxacin, ≤ 0.5 µg/mL; and minocycline, 2 µg/mL. Therapy was switched to ceftazidime on day 21, and the patient was discharged on day 22. The patient received antimicrobial therapy for 7 days. To determine whether *P. otitidis* carried the chromosomally encoded metallo-β-lactamase POM−1, conventional polymerase chain reaction was performed using the primers POM−1_F (5′-CTGCACAGCCACGCCCAC−3′) and POM−1_R (5′-GTCATGCCGCCCAGCTCC−3′) [5]. The strain was found to possess the *POM−1* gene.

## Discussion

*P. otitidis* has been isolated from a wide range of environmental sources, including soil, plants, food products, various aquatic environments, sewage, and public toilets [4], [10]. The closest phenotypic and genotypic *Pseudomonas* spp. to *P. otitidis* was reported to be *Pseudomonas aeruginosa* [5]. Routine biochemical testing cannot reliably distinguish these species; therefore, accurate identification requires matrix-assisted laser desorption ionization time-of-flight mass spectrometry or 16S rRNA gene sequencing.

In our case, the portal of entry remained unknown, as there were no clinical indications of otitis media, respiratory or urinary tract infection, or peritonitis. Several factors may explain the occurrence of bacteremia in this patient. First, neither Charcot’s triad nor Murphy’s sign was observed on hospital day 16. However, given the slight elevation of biliary enzyme levels and the presence of a biliary stent, stent-related cholangitis was considered a possible diagnosis. In addition, as the patient had undergone endoscopic sphincterotomy and papillary balloon dilation, the protective function of the sphincter of Oddi was compromised, potentially leading to retrograde cholangitis. Second, although a definitive diagnosis had not been established, the possibility of intrahepatic cholangiocarcinoma and advanced age may have contributed to modestly impaired host defenses. In previous reports, one case involved bacteremia secondary to cellulitis, while the other two cases were bacteremia of unknown origin [6], [7], [8]. Reports of bacteremia caused by this organism are exceedingly rare, underscoring the need for continued accumulation of clinical data.

POM−1 hydrolyzes penicillins and carbapenems efficiently and exhibits low activity against cephalosporins [5]. Previous studies have shown that *P. otitidis* frequently exhibits intermediate or resistant phenotypes to carbapenems [2], [11] and have also revealed the clinical significance of the carbapenem inoculum effect in *P. otitidis* infections [5]. Based on these characteristics, carbapenems are generally considered suboptimal agents for treating infections caused by this organism. In this case, the isolate, which possess POM−1, demonstrated susceptibility to meropenem based on broth microdilution method, and the patient’s condition improved after antibiotic treatment. Similarly, among IMP-type metallo-β-lactamases, certain variants such as IMP−6 have been described as “stealth types” because they do not consistently confer phenotypic resistance to carbapenem agents [12]. However, carbapenem therapy for carbapenemase-producing organisms, such as those harboring POM−1 or IMP enzymes, is generally not recommended except under specific circumstances. Therefore, careful follow-up is warranted to monitor for potential recurrence of *P. otitidis* infection.

In the present case, antimicrobial therapy was administered for 7 days. A randomized controlled trial demonstrated the noninferiority of a 7-day course to a 14-day course for uncomplicated Gram-negative bacteremia [13]. In addition, an ongoing study is evaluating the safety and efficacy of a 5-day antibiotic therapy course in clinically stable patients with Gram-negative bacteremia [14]. A limitation of this case is the absence of phylogenetic analysis using type strains of closely related species, which would have provided additional support for the identification of this rare bacterial species.

In summary, we report a case of bacteremia caused by *P. otitidis* following endoscopic biliary procedures. Currently, no standard antimicrobial treatment for *P. otitidis* infection has been established. The detection rate of *P. otitidis* in clinical settings remains extremely low, and the organism may be under-recognized. Clinicians should recognize that *P. otitidis* can cause bacteremia and biliary tract infection. Further research is required to clarify the epidemiological and clinical characteristics of *P. otitidis* bacteremia.

## CRediT authorship contribution statement

**Hiroki Yoshikawa:** Writing – review & editing, Conceptualization. **Yasuhisa Hiroshima:** Writing – review & editing, Conceptualization. **Kazufumi Hiramatsu:** Writing – review & editing, Supervision, Conceptualization. **Kosaku Komiya:** Writing – review & editing, Conceptualization. **Ryota Sagami:** Writing – review & editing, Conceptualization. **Takehiro Hashimoto:** Writing – original draft, Visualization, Software, Methodology, Investigation, Formal analysis, Data curation, Conceptualization.

## Ethics declaration

Written informed consent to take part in the study and to publish the article has been obtained from all participants or their legal representatives. The privacy rights of participants have been observed.

This study was performed in compliance with relevant laws, regulatory frameworks and guidelines where the research took place. Ethics committee approval was not required under relevant laws and institutional guidelines. The patient provided informed consent for publication of the report and associated images.

This research follows the CARE guidelines and the CARE checklist.

## Ethical approval

Our institution does not require ethical approval for reporting individual cases.

## Consent for publication

Written informed consent was obtained from the patient for publication of this case report and accompanying images. A copy of the written consent is available for review by the Editor-in-Chief of this journal on request.

## Funding

This research did not receive any specific grant from funding agencies in the public, commercial, or not-for-profit sectors.

## Declaration of Competing Interest

The authors have no conflicts of interest directly relevant to the content of this article.

## Data Availability

The data used and/or analyzed during this study are available from the corresponding author upon reasonable request.
